# Supplementary material for: A Machine Learning Model to Predict Survival and Therapeutic Responses in Multiple Myeloma
Source: Int J Mol Sci. 2023 Apr 3;24(7):6683. doi: 10.3390/ijms24076683 (PMC10095137; doi:10.3390/ijms24076683)
Supplement: Supplementary file 1 [file ijms-24-06683-s001.zip › caption.pdf]

Table S1: Clinical information of BM samples for scRNA sequencing.
Figure S1: Univariate cox regression analysis in the training (A) and validation cohort (B).
Figure S2: The association between UPPRS and patients' responses to PIs in the validation cohort.
Figure S3: (A) Pathways with significant enrichment in different UPPRS groups; (B) The expression of nine UPPGs in the different response group in the validation group.
Figure S4: (A) Kaplan–Meier curve for the UPPRS in ISS = II/III patients in the training cohort; (B) Kaplan–Meier curve for the UPPRS in ISS = II/III patients in the validation cohort; (C) Kaplan–Meier curve for the UPPRS in ISS = II patients in the validation cohort.
Figure S5: The AUCs of ISS, UPPRS, and nomogram predicted 1-year (A) and 2-year (B) survival rates by ROC analysis in the training cohort; the AUCs of ISS, UPPRS, and nomogram predicted 1-year (C) and 2-year (D) survival rates by ROC analysis in the validation cohort. Calibration plots of the nomogram at 1- and 2-years in the training cohort (E), and those treated with PIs in the training cohort (F); Calibration plots of the nomogram at 1,2-year in the validation cohort (G).
Figure S6: (A) UMAP plot of 40675 cells classified by samples; (B) Volcano plot of DEGs between NDMM and RRMM myelomas; (C) Number and strength of communications among cells; (D) Ligand-receptor in the communication of immune cells to two subtypes of myeloma cells. Myeloma cells (H)—high-UPPRS myeloma cells; Myeloma cells (L)—low-UPPRS myeloma cells.
