# Supplementary figures and images for: A Machine Learning Model to Predict Survival and Therapeutic Responses in Multiple Myeloma
Source: Int J Mol Sci. 2023 Apr 3;24(7):6683. doi: 10.3390/ijms24076683 (PMC10095137; doi:10.3390/ijms24076683)

**A**

Training cohort

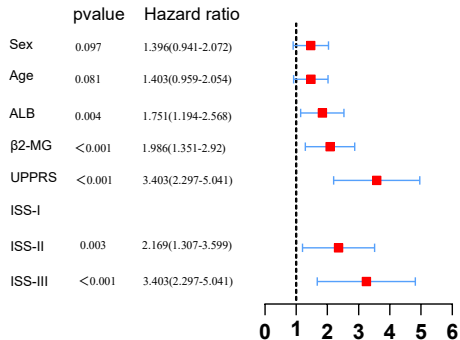**B**

Validation cohort

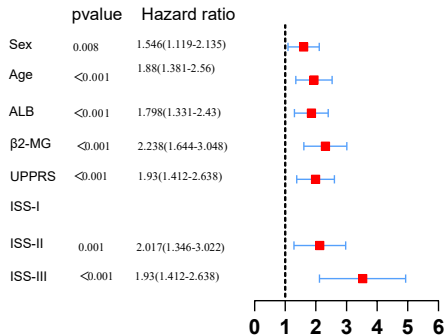

Supplement: Supplementary file 1 [file ijms-24-06683-s001.zip › Figure S1.pdf]

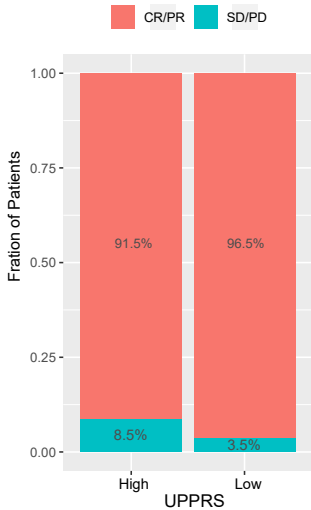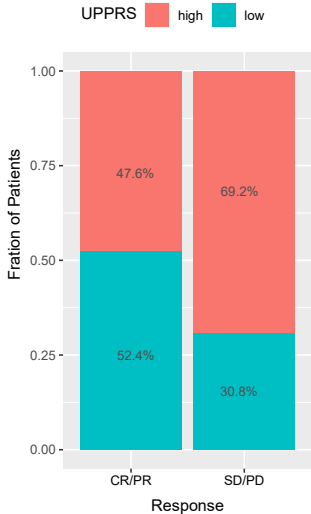

Supplement: Supplementary file 1 [file ijms-24-06683-s001.zip › Figure S2.pdf]

A

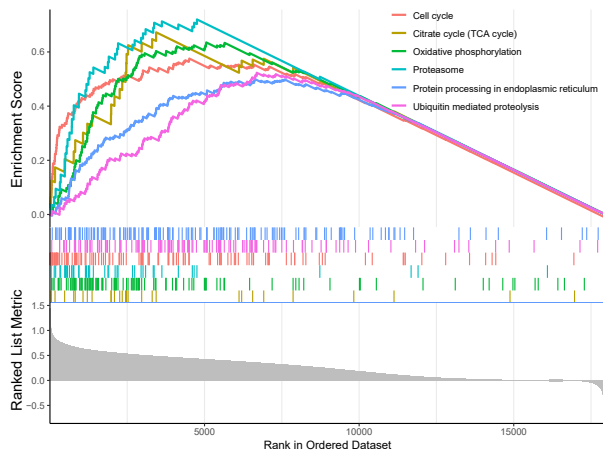

B

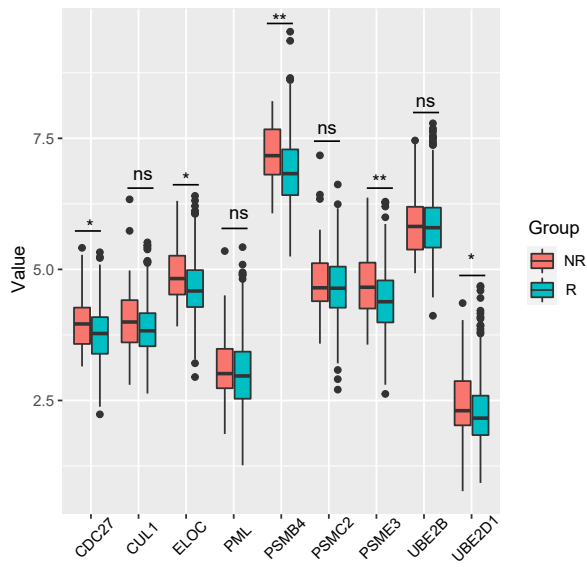

Supplement: Supplementary file 1 [file ijms-24-06683-s001.zip › Figure S3.pdf]

A

Training cohort (ISS=II/III)

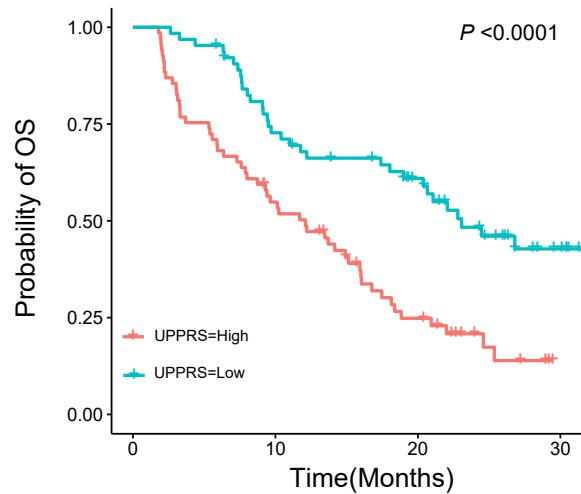

B

Validation cohort (ISS=II/III)

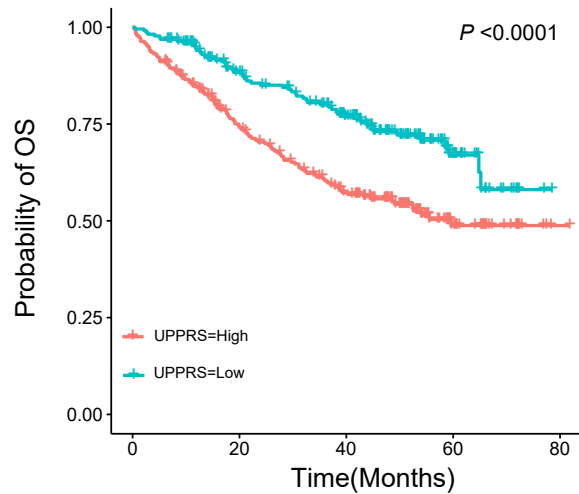

C

Validation cohort (ISS=II)

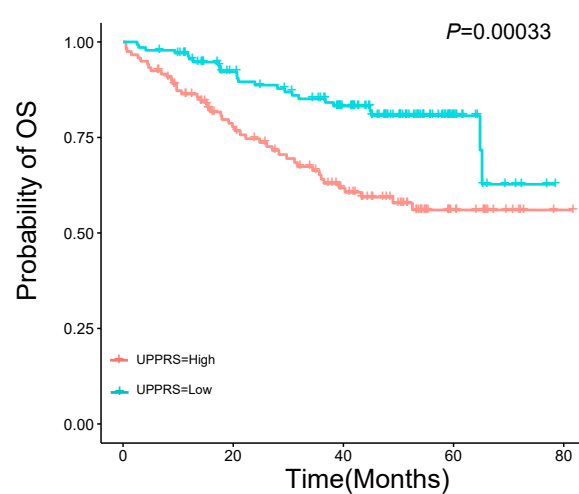

Supplement: Supplementary file 1 [file ijms-24-06683-s001.zip › Figure S4.pdf]

A

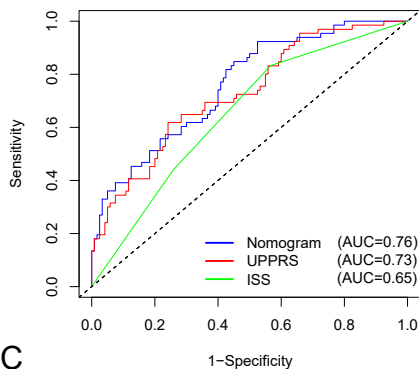

B

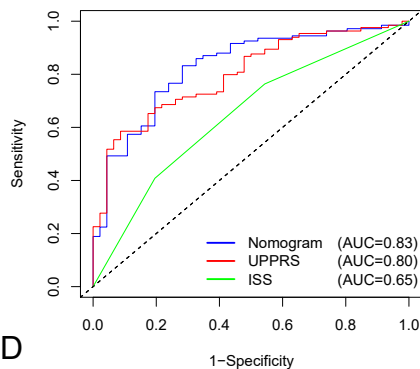

C

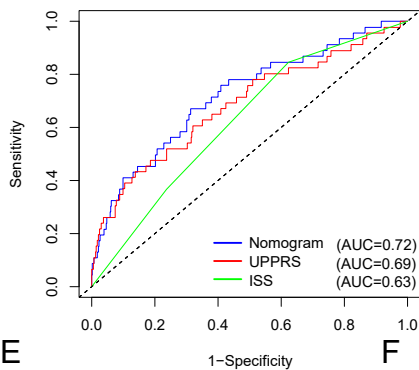

D

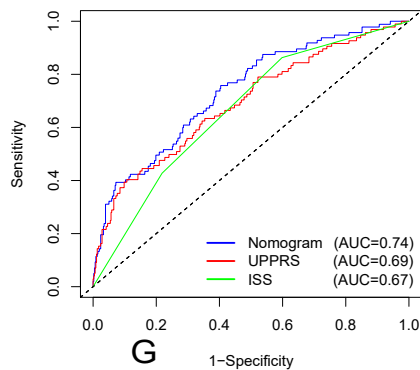

E

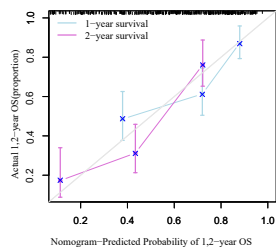

F

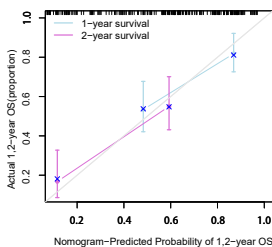

G

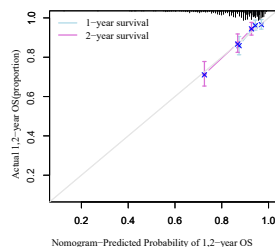

Supplement: Supplementary file 1 [file ijms-24-06683-s001.zip › Figure S5.pdf]

A

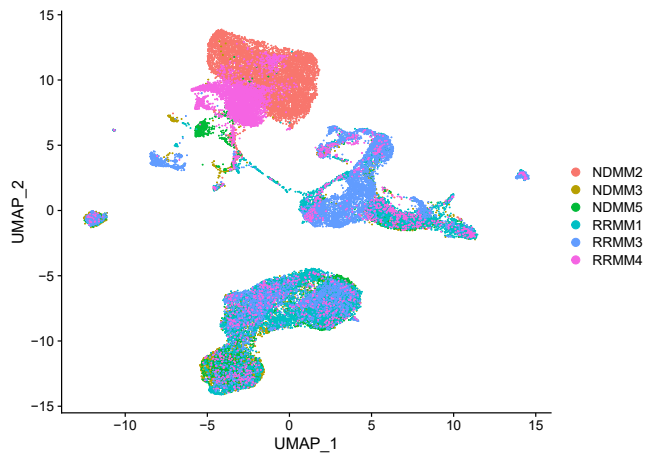

B

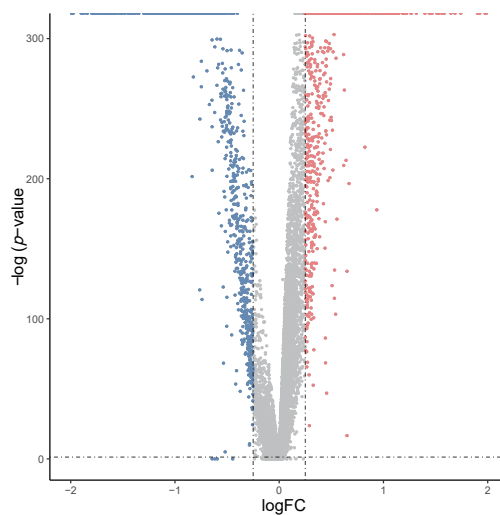

C

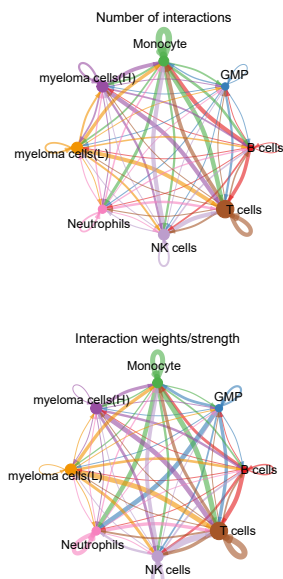

D

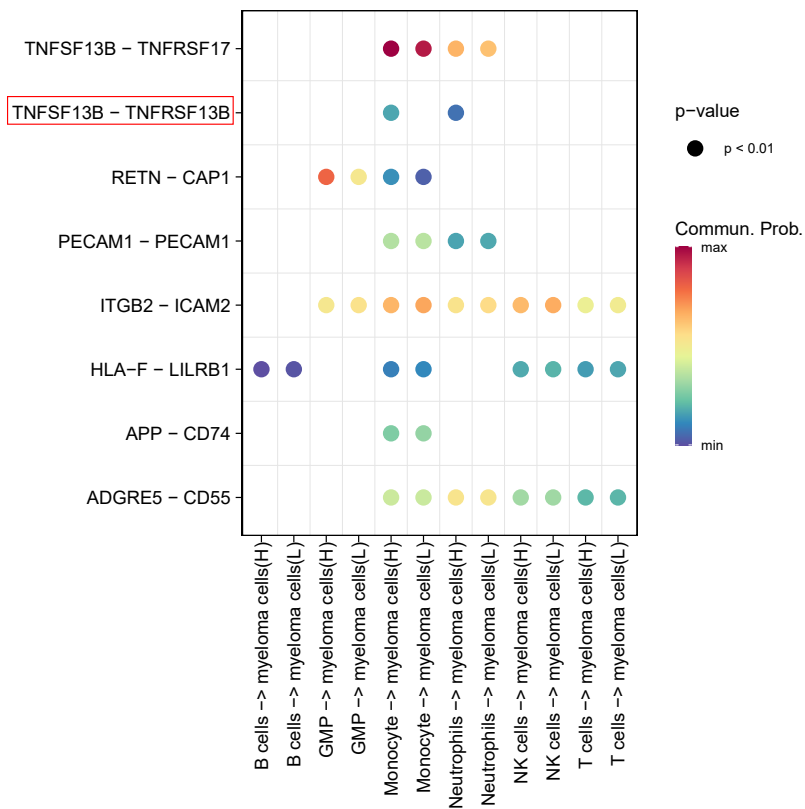

Supplement: Supplementary file 1 [file ijms-24-06683-s001.zip › Figure S6.pdf]
